# Retinoscopy (or Shadow Test) in the Determination of Refraction at One Meter Distance, with the Plane Mirror

**Published:** 1899-06

**Authors:** 


RETINOSCOPY (or Shadow Test) IN THE DETERMINATION OF REFRACTION AT ONE METER DISTANCE, WITH THE PLANE MIRROR. By JAMES THORINGTON, M. D., Adjunct Professor of Diseases of the Eye in the Philadelphia Polyclinic and College for Graduates in Medicine; Assistant Surgeon to Wills' Eye Hospital. Third edition, revised and enlarged. Forty-three illustrations, twelve of which are colored. Duodecimo, pp. eighty-six. Philadelphia: P. Blakiston's Son & Co. 1899.

Retinoscopy is undoubtedly the most reliable subjective test for the errors of refraction that is known. This little book of 86 pages gives a clear description of the method at one meter distance and with the plane mirror. It is now in its third edition, the first having been issued in 1897. This is an indication of its popularity. It is worthy of the favor it has received. A. A. H.

